# Supplementary material for: Mother and newborn skin to skin contact in East Africa: Prevalence and predictors (2019–2023)
Source: PLOS Glob Public Health. 2025 Jun 24;5(6):e0004816. doi: 10.1371/journal.pgph.0004816 (PMC12186949; doi:10.1371/journal.pgph.0004816)
Supplement: Attachment — Submitted filename: Response to Editor comments-.pdf [file pgph.0004816.s003.pdf]

Response to Editor comments

Manuscript ID: PGPH-D-25-00644

Title: Mother and Newborn Skin to Skin Contact in East Africa: Prevalence and Predictors 2019-2023.

Journal: PLOS Global Public Health

Authors

*Alemneh Tadesse Kassie, Tadesse Tarik Tamir, Astewil Moges Bazezew, Agnche Gebremichael, Daniel Asefa Gonete, Alebachew Ferede Zegeye*mailto:alexferede24@gmail.com

Subject: Submission of revised manuscript

Yours sincerely,

Alemneh Tadesse Kassie (on behalf of all authors)

University of Gondar, Gondar, Ethiopia

Dear Editor,

We appreciate the modifications requested for this manuscript. Your comments are encouraging, and they reflect a shared understanding of the clinical significance of our study and its findings. Below, you will find our detailed modifications for [PGPH-D-25-00644] - [EMID:de04b436ed7c1388]. Additionally, the revised manuscript is attached.

Comment:

We noticed some instances of overlapping text with the following previous publications, which need to be addressed:

- https://pubmed.ncbi.nlm.nih.gov/39664655/
- https://www.icsti.org/

In your revision, please ensure all sources are properly cited, including your own works, and that any duplicated text outside the methods section is quoted or rephrased. Further consideration is contingent on addressing these concerns.

Response:

Thank you for pointing out the overlapping text from our previous publication (DOI: 10.3389/fgwh.2024.1471187). We appreciate the opportunity to address these issues and uphold academic integrity. All authors have carefully cited sources, including our own, and we have rephrased or quoted any duplicated text outside the methods section to ensure originality. We are committed to maintaining scholarly standards and have made the necessary adjustments.

Comment:

Please review your reference list to ensure that it is complete and correct. If you have cited papers that have been retracted, please include the rationale for doing so in the manuscript text, or remove these references and replace them with relevant current references. Any changes to the reference list should be mentioned in the rebuttal letter that accompanies your revised manuscript. If you need to cite a retracted article, indicate the article's retracted status in the References list and also include a citation and full reference for the retraction notice.

Response:

Thank you for your comment regarding the reference list. We have thoroughly reviewed it and can confirm that there are no retracted papers included. Additionally, we have not added any new references, as all cited works are current and relevant to our study.

We appreciate your guidance and have ensured the reference list is complete and accurate.

Comment:

Please address the minor comment raised by reviewer 2. Please also carefully check the manuscript for any lingering typos, such as "The first one hours after birth" -> "The first one hour after birth" (or "The first hour after birth").

Response:

Dear Editor, thank you for your comment. I have addressed the minor issue raised by Reviewer 2 and carefully checked the manuscript for any lingering typos. The phrase "The first one hours after birth" has been corrected to "The first hour after birth."

Reviewer comment

Comment:

Reviewer #2: Thank you for addressing all my comments.

I have only one comment left: In line 52, the added statement is not consistent with the results in line 270-271 and in Figure 2.

Response:

Dear Reviewer,

Thank you for your feedback. We have corrected the inconsistency in line 52 to align it with the results presented in lines 270-271 and Figure 2.
