# Supplementary material for: Mother and newborn skin to skin contact in East Africa: Prevalence and predictors (2019–2023)
Source: PLOS Glob Public Health. 2025 Jun 24;5(6):e0004816. doi: 10.1371/journal.pgph.0004816 (PMC12186949; doi:10.1371/journal.pgph.0004816)
Supplement: Attachment — Submitted filename: 2025 PLOS ONE Comments to the authors, STS East Africa.docx [file pgph.0004816.s001.docx]

Review.

This is an interesting manuscript about early skin-to-skin contact in East Africa.

It needs language revisions and some statistic revision (see comment 4b, 7, and 10), and interpretation of results (see comment 8 and 9). There are no line numbers in the manuscript.

Comments:

1. Abstract: Please consider which are the most important results. In the abstract you mention Kenya, Mozambique, and Tanzania. In the main text you mention Rwanda (with the highest prevalence) and Madagascar.
2. Introduction: You have a type error. WHO recommends that skin-to-skin contact should begin within one hour of birth (not at least one hour after).
3. Page 7: All abbreviations in tables (also in the title of the tables) should be explained.
4. Study variables page 8:
   a. The age categories are not the same as in Table 2.
   b. You have listed “Initiation of first breastfeeding within one hour” as an independent variable, but breastfeeding comes after skin-to-skin contact and is not independent of skin-to-skin contact.
5. Results: There is no need for repeating all results from the tables.
6. Table 3:
   a. Please correct the type error in the first Age category.
   b. Community level of poverty: Does “low” mean low level of poverty (or low income, which is the opposite)? Please clarify in the table.
7. Page 16: Do mothers in East Africa initiate breastfeeding first (infant wear clothes) and there after initiate skin-to-skin contact? In my country it is opposite (Infants are placed skin-to-skin immediately after birth and will initiate breastfeeding after 30-90 minutes). Provided infants in your study initiated skin-to-skin contact before breastfeeding, you should re-word your results because the odds of SSC is not dependent of breastfeeding, but the odds of breastfeeding is dependent of SSC. In my opinion the following sentence is wrong: “The analysis revealed that the odds of SSC were 2.24 times higher among newborns who did not receive breastfeeding within the first hour compared to those who did (AOR = 2.24, 95% CI: 1.86, 2.69).”
8. Page 16, your sentence: “Additionally, newborns of mothers with only primary education had 1.60 times higher odds of SSC compared to those whose mothers had no formal education or secondary education and above (AOR = 1.60, 95% CI: 1.24, 2.07).” In the variable you have one category as reference, that means you are comparing only to this category (not to “secondary education and above”).
9. Same problem with antennal care visits: You are only comparing to “<4 visits”, not to “no visits”.
10. Regression analyses: Please give a rational in the methods when you choose the middle category as reference instead of highest or lowest (Antenatal care visits, and House hold wealth index).
11. Discussion page 20: Your sentence: “Health service coverage, the quality of maternal and child healthcare, and economic and health policies in Vietnam and the U.S. are generally more robust than those in East African countries.” Please explain why a free of charge intervention like skin-to-skin contact could be influenced by economic.
12. The conclusion is very good. Please also consider to include the role of the health care providers in the discussion, not just in the conclusion.
