# Supplementary material for: Mother and newborn skin to skin contact in East Africa: Prevalence and predictors (2019–2023)
Source: PLOS Glob Public Health. 2025 Jun 24;5(6):e0004816. doi: 10.1371/journal.pgph.0004816 (PMC12186949; doi:10.1371/journal.pgph.0004816)
Supplement: Attachment — Submitted filename: Response to Editor comments.docx [file pgph.0004816.s002.docx]

**Response to Editor comments**

**Manuscript ID:** PGPH-D-25-00644
**Title:** Mother and Newborn Skin to Skin Contact in East Africa: Prevalence and Predictors 2019-2023.
**Journal:** PLOS Global Public Health

## **Authors**

*Alemneh Tadesse Kassie, Tadesse Tarik Tamir, Astewil Moges Bazezew, Agnche Gebremichael, Daniel Asefa Gonete, Alebachew Ferede Zegeye*

**Subject:** Submission of revised manuscript

Yours sincerely,

**Alemneh Tadesse Kassie** **(on behalf of all authors)**

University of Gondar, Gondar, Ethiopia

**Dear Editor,**

We appreciate the modifications requested by the editor for this manuscript. The comments are encouraging, and the review reflects a shared understanding of the clinical importance of our study and its results. Below, you will find our detailed modifications for [PGPH-D-25-00644] - [EMID:de04b436ed7c1388]. Additionally, please find the attached file of the revised manuscript.

**Comment:** We noticed you have some minor occurrence of overlapping text with the following previous publication(s), which needs to be addressed:

<https://pubmed.ncbi.nlm.nih.gov/39664655/> <https://www.icsti.org/>

In your revision ensure you cite all your sources (including your own works), and quote or rephrase any duplicated text outside the methods section. Further consideration is dependent on these concerns being addressed. Comments from PLOS Editorial Office: We note that one or more reviewers has recommended that you cite specific previously published works. As always, we recommend that you please review and evaluate the requested works to determine whether they are relevant and should be cited. It is not a requirement to cite these works. We appreciate your attention to this request

**Response:** Thank you for highlighting the need for revisions regarding the overlapping text from our previous publication (DOI: .org/10.3389/fgwh.2024.1471187). We appreciate the opportunity to address these concerns and uphold academic integrity. All authors have cited sources diligently, including our own, and we have rephrased or quoted any duplicated text outside the methods section to ensure originality. We are committed to maintaining scholarly standards and have made the necessary adjustments. Please let us know if you have any further comments of the manuscript.

**Reviewer comments and Authors response’s**

**Reviewer #1:**

**Comment:**

1. The time of the study should be stated in the title.

**Response:** Dear Reviewer, thank you for the suggestion; I have included it.

**Comment:**
2. The title and purpose should be the same.
**Response:** Dear Reviewer, thank you; I have revised them to align as suggested.

**Comment:**
3. Explain more about Methods (tools). The purpose of the study should be stated at the end of the introduction.
**Response:** Dear Reviewer, thank you; I have made the necessary corrections.

**Comment:**
4. Explain about tools "validity and reliability."
**Response:** Dear Reviewer, thank you; I have incorporated details on validity and reliability in the methods section based on your recommendation.

**Comment:**
5. Discussion is lengthy.
**Response:** Dear Reviewer, thank you; I have shortened the discussion as suggested.

**Comment:**
You can use these articles in your introduction and discussion:

1. Khadivzadeh T, Karimi FZ, Tara F. Effects of early mother-neonate skin-to-skin contact on the duration of the third stage of labor: A randomized clinical trial. The Iranian Journal of Obstetrics, Gynecology and Infertility, 2018; 21(2): 23-29. doi: 10.22038/ijogi.2018.10704
2. Karimi ZF, Abdollahi M, Khadivzadeh T, Yas A. Investigating the Effect of Kangaroo Mother Care on Maternal-Infant Attachment: A Systematic Review and Meta-Analysis Study, Current Women’s Health Reviews 2024; 20(2): e280223214099. <https://dx.doi.org/10.2174/1573404820666230228093256>

**Response:** Dear Reviewer, thank you for recommending these valuable sources; we have reviewed them and updated our manuscript accordingly.

**Reviewer #2:**

**Comment:**

This is an interesting manuscript about early skin-to-skin contact in East Africa. It needs language revisions and some statistical revisions (see comments 4b, 7, and 10), and interpretation of results (see comments 8 and 9). There are no line numbers in the manuscript.

**Response:** Dear Reviewer, thank you for your feedback. We have made language and statistical revisions, improved the interpretation of results, and incorporated line numbers into the manuscript as you advised.

**Comment:**

Abstract: Please consider which are the most important results. In the abstract, you mention Kenya, Mozambique, and Tanzania. In the main text, you mention Rwanda (with the highest prevalence) and Madagascar.

**Response:** Dear Reviewer, thank you for your observation. In the abstract's results section, we highlighted the countries that are significantly associated, but we acknowledge that the country with the highest prevalence should be included. We have made this adjustment in the recommendations and conclusion sections of the abstract.

**Comment:**

**2.** Introduction: You have a typo. WHO recommends that skin-to-skin contact should begin within one hour of birth (not at least one hour after).

**Response:** Dear Reviewer, you are correct, and we have corrected this as per your direction. Thank you.

**Comment:**

**3.** Page 7: All abbreviations in tables (also in the title of the tables) should be explained.

**Response:** Dear Reviewer, thank you for your valuable advice. We have added explanations for all abbreviations as you requested.

**Comment:**

**4.** Study variables page 8:

**a.** The age categories are not the same as in Table 2.

**Response:** Dear Reviewer, thank you for your comment. We have corrected the age categories to ensure consistency with Table 2.

**Comment:**
b. You have listed “Initiation of first breastfeeding within one hour” as an independent variable, but breastfeeding comes after skin-to-skin contact and is not independent of skin-to-skin contact.

**Response:** Thank you for your insightful feedback. We appreciate your careful review of our variables. While it is true that breastfeeding commonly follows skin-to-skin contact, our intention in listing “Initiation of first breastfeeding within one hour” as an independent variable was to assess its specific predictors and associations, separate from—but still in relation to—skin-to-skin contact.

We recognize that early breastfeeding and skin-to-skin contact are closely linked, often occurring sequentially and reinforcing each other’s benefits. However, given variations in hospital practices and maternal circumstances, early breastfeeding does not always immediately follow skin-to-skin contact. Therefore, we have analyzed it as an independent variable to understand its distinct determinants within the broader context of maternal and newborn care. We truly appreciate your perspective and are open to refining our approach if necessary to enhance clarity and accuracy. Thank you again for your valuable input!

**Comment:**
5. Results: There is no need for repeating all results from the tables.

Response: Dear Reviewer, thank you for your recommendation. We have revised the results section to be more selective, following your guidance.

**Comment:**
6. Table 3:
a. Please correct the type error in the first Age category.

**Response:** Dear Reviewer, thank you; we have corrected it as you requested.
b. Community level of poverty: Does “low” mean low level of poverty (or low income, which is the opposite)? Please clarify in the table.

**Response:** Dear Reviewer, thank you for pointing out this clarity issue. “Low” refers to a low level of poverty, and we have clarified this in the table as you requested.

**Comment:**
7. Page 16: Do mothers in East Africa initiate breastfeeding first (infant wear clothes) and there after initiate skin-to-skin contact? In my country it is opposite (Infants are placed skin-to-skin immediately after birth and will initiate breastfeeding after 30-90 minutes). Provided infants in your study initiated skin-to-skin contact before breastfeeding, you should re-word your results because the odds of SSC is not dependent of breastfeeding, but the odds of breastfeeding is dependent of SSC. In my opinion the following sentence is wrong: “The analysis revealed that the odds of SSC were 2.24 times higher among newborns who did not receive breastfeeding within the first hour compared to those who did (AOR = 2.24, 95% CI: 1.86, 2.69).”

**Response:** Thank you for your thoughtful comment. We acknowledge that the sequence of skin-to-skin contact (SSC) and breastfeeding may vary across regions and healthcare practices. In our study setting, SSC is generally initiated immediately after birth, often accompanied by early breastfeeding. However, we recognize that its implementation is inconsistent across deliveries.

We appreciate your clarification regarding the relationship between SSC and breastfeeding initiation. Based on this, we agree that the wording of our results should accurately reflect the dependency of breastfeeding on SSC rather than vice versa. Accordingly, we have revised our statement to ensure clarity and precision:

“In this study, the odds of initiating breastfeeding within the first hour were 2.24 times higher among newborns who experienced SSC compared to those who did not (AOR = 2.24, 95% CI: 1.86, 2.69).’’ We are grateful for your valuable input and remain committed to refining our findings for accuracy. Thank you for your constructive feedback.

**Comment:**
8. Page 16, your sentence: “Additionally, newborns of mothers with only primary education had 1.60 times higher odds of SSC compared to those whose mothers had no formal education or secondary education and above (AOR = 1.60, 95% CI: 1.24, 2.07).” In the variable you have one category as reference, that means you are comparing only to this category (not to “secondary education and above”).

**Response:** Thank you for your keen observation! We appreciate the clarification and have edited the sentence accordingly. Your feedback is invaluable in ensuring accuracy. Many thanks!

**Comment:**
9. Same problem with antennal care visits: You are only comparing to “<4 visits”, not to “no visits”.

**Response:** Thank you for your keen observation! We appreciate the clarification and have edited the sentence accordingly. Your feedback is invaluable in ensuring accuracy. Many thanks!

**Comment:**
10. Regression analyses: Please give a rational in the methods when you choose the middle category as reference instead of highest or lowest (Antenatal care visits, and House hold wealth index).

**Response:** Thank you for your insightful question! We selected the middle category as the reference group for antenatal care visits and household wealth index to provide a balanced comparison and better capture the effects of both lower and higher categories.

By using the middle category as the reference, we aim to assess deviations in both directions—whether fewer antenatal care visits or a lower/higher wealth index influence skin-to-skin contact (SSC) outcomes. This choice allows us to avoid potential biases that may arise if the highest or lowest category is used as the reference, as extreme values could distort interpretations. We appreciate your feedback and have refined the explanation in the methods section to clarify this rationale. Thank you again!

**Comment:**
11. Discussion page 20: Your sentence: “Health service coverage, the quality of maternal and child healthcare, and economic and health policies in Vietnam and the U.S. are generally more robust than those in East African countries.” Please explain why a free of charge intervention like skin-to-skin contact could be influenced by economic.

**Response:** Thank you for your thoughtful question. While SSC is a cost-free intervention, its implementation is still influenced by economic factors. Healthcare infrastructure, staffing, and training—often dependent on financial resources—impact how consistently SSC is practiced. Additionally, economic policies shape healthcare priorities, influencing investment in maternal and newborn care. In lower-income settings, hospital constraints, early discharges, and limited awareness may hinder SSC adoption. Moreover, cost-free practices like SSC are often overlooked in resource-limited settings due to competing priorities. We appreciate your insight and will refine our discussion accordingly.

**Comment:**
12. The conclusion is very good. Please also consider to include the role of the health care providers in the discussion, not just in the conclusion.

**Response:** Thank you for your comment! We have corrected it and incorporated the role of healthcare providers into the discussion section as well. We appreciate your valuable feedback!
